# The impact of emotional abuse on Internet addiction in Chinese children: the sequential mediation roles of emotional dysregulation and regulatory emotional self-efficacy

**DOI:** 10.3389/fpsyg.2025.1517489

**Published:** 2025-02-05

**Authors:** Wang Liu, Jie Huang, Yu-Ling Li, Xiang Gao, Zi-Yi Xu, Yong-Hui Li

**Affiliations:** ^1^Department of Psychology, Hunan First Normal University, Changsha, China; ^2^Hunan Key Laboratory of Children’s Psychological Development and Brain, Changsha, China; ^3^Key Laboratory of Mental Health, Institute of Psychology, Chinese Academy of Sciences, Beijing, China; ^4^Department of Psychology, University of Chinese Academy of Sciences, Beijing, China

**Keywords:** emotional abuse, Internet addiction, emotional dysregulation, regulatory emotional self-efficacy, emotional terrorizing, emotional neglect

## Abstract

**Background:**

In China, emotional abuse and Internet addiction are increasingly prevalent among children, with significant negative effects on their development. Previous research has identified childhood emotional abuse as a major risk factor for Internet addiction in both adolescents and adults. However, the immediate impact of emotional abuse on children’s Internet addiction within Chinese culture remains unclear.

**Objective:**

This study aimed to investigate the impact of emotional abuse on Internet addiction through emotional dysregulation and regulatory emotional self-efficacy in Chinese children.

**Methods:**

A sample of 556 fourth to sixth grade primary school students (girls, 46.6%) with an average age of 10.30 ± 0.93 years were recruited from a central province in China. Participants completed the paper-and-pencil survey on emotional abuse, emotional dysregulation, regulatory emotional self-efficacy, and Internet addiction. The hypothesized model was tested using Mplus 8.3 with 5,000 bootstrap samples.

**Results:**

Emotional abuse positively predicts Internet addiction in children; emotional dysregulation and regulatory emotional self-efficacy mediate this relationship independently, and have chain-mediating effects in this relationship.

**Conclusion:**

Emotional abuse impacts children’s Internet addiction through the development of emotional regulation and self-perception of their abilities in emotional regulation. The findings offer potential strategies for preventing children’s Internet addiction.

## Highlights

- Emotional abuse is a risk factor of Internet addiction in children.
- Emotional dysregulation and regulatory emotional self-efficacy mediated the relationship between emotional abuse and children's Internet addiction both independently and sequentially.
- Building confidence in emotional abilities can prevent children from developing Internet addiction.

## 1 Introduction

Internet addiction is a growing problem in children worldwide, particularly in countries with easier access to computers and the Internet. In China, the Internet penetration rate among primary school students has reached up to 92.1% by 2020 (CNNIC, 2021) and 95.1% by 2022 (CNNIC, 2023). The wide availability of mobile applications provides more chances for children to observe or engage in short videos, online games, and social networking, potentially increasing their susceptibility to Internet addiction. Emotional abuse, one of the most prevalent maltreatment types in childhood (Huang et al., 2023), has been shown to be an important risk factor of Internet addiction in adolescents (Sun et al., 2019) and adults (Arslan, 2017). When children are exposed to repeated emotional abuse from their parents, such as terrorizing, neglecting, derogation, and interference (Kairys and Johnson, 2002; Pan et al., 2010), they may internalize the negative emotions associated with abusive experiences and resort to the Internet as a means of self-consolation and avoidance. Although emotional abuse is a universal problem worldwide, individuals in Chinese culture may perceive and interpret emotional abuse in a different way from those in western culture. In particular, emotional abuse may be more prevalent and more challenging to recognize, acknowledge, and address within Chinese cultural contexts. For this, the impact of emotion abuse on Internet addiction in Chinese children and its underlying mechanism need to be studied.

### 1.1 Emotional abuse and Internet addiction in Chinese children

Internet addiction is defined as compulsive, pathological, or problematic Internet use (Young, 1999), and it is closely associated with various emotional disorders such as depression, anxiety, and stress (Xue et al., 2023), cognitive impairments (Ioannidis et al., 2019), sleep disturbances (Lu et al., 2023), and decreased motivation for achievement (Sayegh et al., 2021). Given the alarming prevalence of Internet addiction in Chinese children and its severe physiological, mental and social consequences, there is an urgent need to identify the risk factors of Internet addiction, so that more effective interventions can be developed to mitigate its impact on children. It is known that school-aged children are particularly vulnerable to Internet addiction in China (Dong et al., 2020), especially those aged 9–12 years. Due to reduced self-control and lower academic aspirations (Eccles and Roeser, 2011), Internet addiction is more prevalent among children aged 9 to 12 years—corresponding to primary school students in grades four through six in China—compared to adolescents aged 13–14 years, who are in grades seven through nine (Li et al., 2022).

Emotional abuse may be an important risk factor of Internet addition in Children. Emotional abuse can take various forms, including threatening, frightening, neglecting, blaming, discriminating, humiliating, or ridiculing (Cui and Liu, 2020). Such abuse is likely to adversely affect a child’s physical and mental health, as well as their overall development, potentially leading to both internalizing and externalizing disorders (Cui and Liu, 2020; Simon et al., 2024; Schlensog-Schuster et al., 2024). The impact of emotional abuse on emotional development is particularly pronounced (Cui and Liu, 2020). A systematic review of 25 studies has highlighted that emotional neglect, in particular, has a profound impact on emotional regulation, underscoring the crucial role of emotional abuse in the emotional development of children (Simon et al., 2024). Compared to physical or sexual abuse (Dye, 2020), emotional abuse among childhood maltreatment is a more significant predictor of various negative emotions in children, such as loneliness (Chen and Qin, 2020), anxiety (Shojaati et al., 2021; Chen and Qin, 2020), and depression (Tian et al., 2023). Young children may be especially susceptible to emotional abuse from their parents for three reasons. First, young children are at a critical stage of self-consciousness development (Nwauzoije et al., 2023), which may amplify the negative effects of emotional abuse. Second, children may be less capable of coping with emotional abuse because of limited cognitive development (Kumari, 2020). Third, children are very likely to actively internalize the negative message conveyed by emotional abuse of their parents because they have to cling to their parents from whom they are seeking protection and support. Thus, young children are more likely to become victims of emotional abuse, which can result in the accumulation of negative emotions and hinder their ability to regulate these emotions effectively.

The Internet Compensation Theory suggests that individuals experiencing negative emotions and lacking effective emotional coping strategies may turn to the Internet as a compensatory mechanism to manage their accumulated negative feelings, potentially leading to Internet addition (Valkenburg and Peter, 2009). Consequently, childhood emotional abuse, as a form of childhood maltreatment, may serve as a significant predictor of Internet addition in children. Previous research has indicated that emotional abuse within families is a key factor in the development of smartphone addiction among adolescents (Sun et al., 2019) and Internet addition among adults (Arslan, 2017). However, despite this, there has been limited focus on examining the relationship between emotional abuse and Internet addition in children. Moreover, retrospective assessments of emotional abuse during adolescence or adulthood, compared to childhood, may introduce recall bias, as individuals may have difficulty accurately remembering or interpreting experiences from their earlier years (Danese, 2020; Yu et al., 2024). Exploring the impact of emotional abuse during its early occurrence in childhood on Internet addiction helps to gain a more accurate understanding of the relationship between emotional abuse and Internet addiction.

In spite of the general acknowledgement of the negative impact of emotional abuse, it is perceived and interpreted differently by individuals from different cultural backgrounds. In China, the understanding of emotional abuse may be influenced by specific elements of Chinese culture, such as the cultural norms of collectivism, traditional values like filial piety and respect for authority, and public attitudes that stigmatize mental health issues. These cultural factors may contribute to the higher prevalence of emotional abuse, making it more difficult to detect, recognize, and address appropriately. Firstly, different from western parents, a large majority of Chinese parents tend to adopt an authoritarian parenting style within a collectivist culture (Ning, 2022). In a collectivist culture, many Chinese parents subscribe to the educational philosophy that “without discipline, there is no success,” believing that strict and demanding parenting methods are essential for their children’s development (Liu et al., 2024). While some parents employ physical punishment, others, though not physically violent, use criticism or verbal reprimands to enforce strict discipline. However, these non-physical punitive parenting behaviors may involve actions that constitute emotional abuse—such as terrorizing, neglecting, belittling, and excessive interference—in an effort to enforce obedience, based on the belief that these methods are beneficial for children. For instance, when children express ideas that differ from their parents’ views, parents might use threats such as, “If you do not comply, I’ll abandon you,” to coerce obedience. This approach can instill a fear of losing parental support. Similarly, derogatory remarks like, “Why cannot you do anything right?” can lead to self-doubt and depression in children. Additionally, excessive interference can impede the development of the child’s abilities and diminish their self-confidence. As parents often fail to recognize the emotional toll that such practices can inflict on their children (Liu et al., 2024), behaviors that are considered emotionally abusive in Western contexts may be seen as part of normal parenting in China. This increases the likelihood that Chinese parents may unconsciously engage in emotional abuse during the parenting process. Research estimates that one in five Chinese has experienced emotional abuse (Fang et al., 2015; Ji and Finkelhor, 2015).

Secondly, filial piety and respect for authority are core traditional values within Chinese culture (Bedford and Yeh, 2019; Fu et al., 2007), which may make children more inclined to obey their parents. The saying “Filial piety is the foremost of all virtues” reflects the deep-rooted belief in China that children should unconditionally follow their parents’ decisions and ideas. Additionally, Chinese society places significant importance on respect for authority, which further strengthens the expectation that children will adhere to their parents’ wishes and not challenge their authority. This cultural dynamic creates a unique situation where children, in their desire to be filial, may comply with various forms of emotional abuse from the parents, such as intimidation, neglect, belittling, or excessive control. In these circumstances, children may suppress their own emotions, believing they should accept how their parents treat them. Even if they are aware of the emotional abuse, they may feel powerless to resist or challenge their parents’ authority. This is in contrast to the more egalitarian norms seen in many Western cultures, where children are more likely to question or oppose authority (Fuligni, 1998). Therefore, for children in China, experiences of emotional abuse are often ignored and repressed, making it difficult for both the children and parents to recognize the harm.

Thirdly, in Chinese culture, there may be a stronger stigma attached to acknowledging mental health issues, as it can conflict with the ideal of family harmony and the importance of maintaining social face (Yang et al., 2007). The saying “Family scandals should not be made public” reflects this cultural tendency, which often results in emotional abuse or mental health problems arising from such abuse being ignored or concealed within Chinese families. Moreover, China currently lacks a comprehensive-legal framework for identifying and addressing childhood abuse including emotional abuse (Wang et al., 2020). Therefore, emotional abuse and the mental health issues it causes become more covert, harder to detect, and challenging to address appropriately, both within families and in the broader public sphere. Overall, these cultural factors shape the perception of emotional abuse, leading many to view it as common, ignorable, and something that should be concealed. Thus, investigating the impact of emotional abuse on Internet addiction in Chinese children would provide evidence to support the generalizability of previous studies to different cultures.

### 1.2 Emotional dysregulation and regulatory emotional self-efficacy as mediators

Although there is some theoretical and empirical evidence that emotional abuse is a significant risk factor of Internet addiction in children, the underlying mechanism linking emotional abuse and Internet addiction is yet to be elucidated. The Person-Affect-Cognition-Execution (I-PACE) model may explain this mechanism, which provides a framework for understanding the psychological and neurobiological processes underlying the development and persistence of Internet addiction, such as those associated with gaming, gambling, pornography, online shopping, and social networking (Brand et al., 2016). The I-PACE model illustrates the process of Internet addiction by defining predisposing factors, such as environmental influences that increase vulnerability to specific Internet-use disorders, and by summarizing the roles of variables as moderators and mediators in the addiction process, while differentiating between environmental aspects, individual reactions, and cognitive factors involved in addictive behaviors (Brand et al., 2019). As the I-PACE model suggested, negative early childhood experiences are one of the core predisposing variables contribute to Internet addiction (Brand et al., 2019). Additionally, in line with the I-PACE model, negative early childhood experiences can influence the coping styles and cognitive responses of abused ones substantially which then become the trigger of Internet addiction (Brand et al., 2019). This suggests that emotional abuse, as a form of negative early childhood experience, may accelerate Internet addiction in children by sequentially altering their coping styles and cognitive responses.

Emotional dysregulation, resulting in negative consequence for individuals in coping with emotions, is used to describe an individual’s inabilities to manage and respond to emotions in a socially adaptive manner (Sáez-Suanes et al., 2023). According to the I-PACE model, emotionally abused children are prone to developing emotional dysregulation, potentially contributing to the development of Internet addiction (Warmingham et al., 2019). Children exposed to emotional abuse often experience heightened negative emotions, which can impair their emotional regulation abilities (Kim et al., 2023). Children who endure emotional abuse are more likely to have their emotional expressions disregarded, downplayed, or penalized by adults (Eisenberg et al., 1996). Consequently, these children may suppress or restrain their emotional expression and instead adopt maladaptive emotion regulation strategies, including rumination, suppression, or avoidance (Krause et al., 2003; Kim et al., 2023). Additionally, emotionally abused children may receive inadequate parental guidance on emotional regulation (Lippard and Nemeroff, 2020), and even worse, they may model their parents’ negative behaviors and coping strategies and therefore be more susceptible to emotional dysregulation (Morris et al., 2007). Furthermore, the Internet Compensation Theory posits that individuals with emotional dysregulation may develop Internet addiction as a compensatory mechanism to avoid accumulated negative emotions (Valkenburg and Peter, 2009). Moreover, there is substantial evidence for the relationship between emotional dysregulation and the development and persistence of Internet addiction in adolescents and adults (Mo et al., 2018; Gioia et al., 2021; Azizi et al., 2024). Young Internet users are particularly susceptible to Internet addiction due to their inabilities to effectively regulate emotions (Gioia et al., 2021). Taken together, emotional dysregulation likely plays a significant mediating role in the relationship between emotional abuse and Internet addiction in children.

Regulatory emotional self-efficacy is defined as an individual’s confidence in effectively managing, understanding, discerning, and regulating his or her emotions (Bandura et al., 2003), and it may represent an important self-cognition of one’s emotional regulation capabilities (Caprara et al., 2003). According to the I-PACE model, emotional abuse has the potential to alter children’s cognitive responses behave in specific ways; e.g. urges to play online games or view pornography (Brand et al., 2019). Emotional abuse may lead children to develop a cognitive bias toward negative emotions, making them more sensitive to negative feelings (Lee and Hoaken, 2007). They may either avoid or indulge in these negative emotions, which can result in a diminished belief in their ability to manage these feelings effectively. In response, they may turn to specific online activities as a way to either escape from or alleviate their emotional distress (Brand et al., 2019). This suggests that emotional abuse might impair the regulatory emotional self-efficacy of abused children, thereby increasing their susceptibility to Internet addiction. In support of this prediction, research has found that emotional neglect, a type of emotional abuse, negatively impacts regulatory emotional self-efficacy in adults (Xie et al., 2022). There is also a strong negative correlation between the general self-efficacy belief and Internet addiction in college students (Berte et al., 2021). These findings indicate that emotional abuse may predict lower regulatory emotional self-efficacy, which in turn may predict higher Internet addiction in children.

### 1.3 The serial mediating role of emotional dysregulation and regulatory emotional self-efficacy

Emotional dysregulation may serve as a negative predictor of emotional regulation self-efficacy. On one hand, the theory of learned helplessness suggests that repeated failures in emotional regulation can lead to feelings of powerlessness (Maier and Seligman, 1976), which in turn undermines confidence in one’s emotional regulation abilities. On the other hand, emotional dysregulation can trigger negative emotional states, such as depression, which further diminishes an individual’s belief in their ability to successfully regulate their emotions (Mesurado et al., 2018). Additionally, according to Social Cognitive Theory, individuals’ cognition is shaped by their own experiences and feedback, which in turn influences their behavior (Bandura, 1999). When children experience difficulties in emotional regulation due to emotional abuse, their perception of their ability to regulate emotions may shift. They may come to believe that they are incapable of effectively managing their emotions, leading to low regulatory emotional self-efficacy. As a result, they may turn to the Internet as a coping mechanism to alleviate their emotional distress. This process suggests that changes in emotional regulation ability can lead to corresponding shifts in individuals’ cognitive perceptions of their own abilities. Taken together, emotional dysregulation and emotional regulation self-efficacy could potentially function as sequential mediators in the relationship between emotional abuse and Internet addiction in children.

### 1.4 The current study

**Figure 1:**
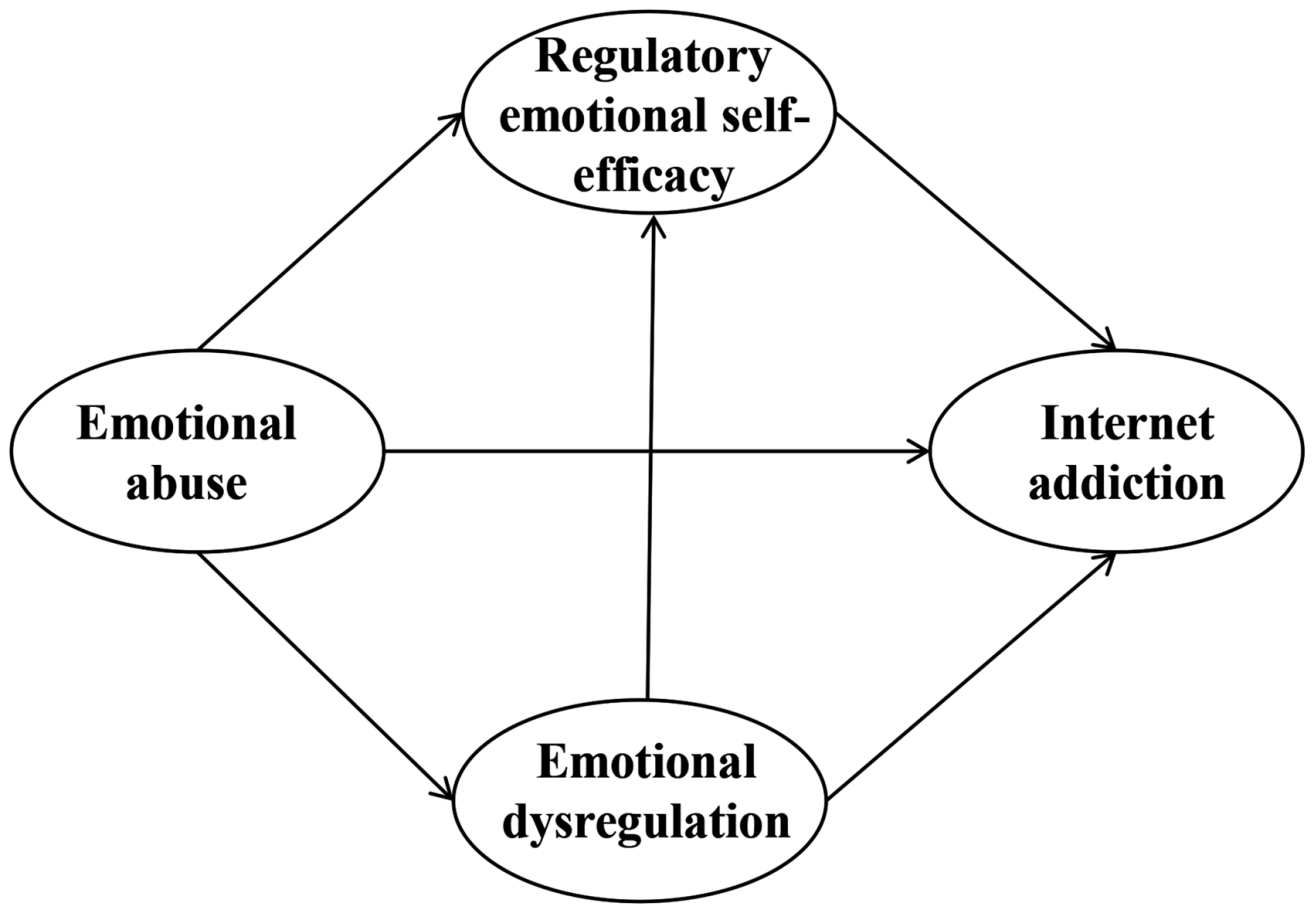
The proposed sequential mediation model of the relationship between emotional abuse and internet addiction in Chinese children.

How emotional abuse impacts Internet addiction in Chinese children remain unclear. Given the serious consequences of emotional abuse and Internet addiction in Chinese children, a sequential mediation model was developed based on the I-PACE model and existing empirical evidence. This model seeks to investigate the association between emotional abuse and Internet Addiction in children. Three hypotheses are made: (1) emotional abuse positively predicts Internet addiction; (2) emotional abuse indirectly predicts Internet addiction through the independent mediation effects of emotional dysregulation and regulatory emotional self-efficacy; and (3) emotional abuse indirectly predicts Internet addiction through the chain-mediation effects of emotional dysregulation and regulatory emotional self-efficacy. Figure 1 illustrates the conceptual model proposed in this study.

## 2 Materials and methods

### 2.1 Participants and procedure

A total of 570 grade four to six primary school students were recruited from three schools in a central province of China and 556 returned valid responses, resulting in a valid response rate of 97.54%. The mean age of the participants was 10.30 years (*SD* = 0.93). Of the 556 participants, 46.6% were girls and 53.4% were boys, which was consistent with the sex ratio of primary school students in China a general; 31.3% were in grade 4, 34.7% were in grade 5, and 34.0% were in grade 6. Regarding parental education, 41.0% of fathers held a bachelor’s degree, while 40.1% of mothers did not achieve a bachelor’s degree. The detailed demographic information is presented in Table 1. In this study, the Monte Carlo Power Analysis for Indirect Effects was performed to establish the necessary sample size (Schoemann et al., 2017). The results show that a minimum of 275 participants would be required to achieve a robust statistical power of 90% in a sequential mediation model. Thus, the sample size is sufficiently large in this study.

**Table 1:** Demographics of the participants (*N* = 556).

Variable	*N* (%)
Gender	
Boys	297 (53.4)
Girls	259 (46.6)
Grade	
The fourth grade	174 (31.3)
The fifth grade	193 (34.7)
The sixth grade	189 (34.0)
Only child	
Yes	401 (72.1)
No	155 (27.9)
Paternal education level	
High school and below	201 (36.2)
Bachelor’s degree	228 (41.0)
Above bachelor’s degree	127 (22.8)
Maternal education level	
High school and below	223 (40.1)
Bachelor’s degree	208 (37.4)
Above bachelor’s degree	125 (22.5)

This study was approved by the Ethical Committee of Science and Technology of our university (No. 202201) and complied with the Helsinki Declaration. The data were collected between February and May 2022. The participants and their parents signed informed consent forms. Children completed a questionnaire covering their demographic characteristics, experiences of emotional abuse, emotional dysregulation, regulatory emotional self-efficacy, and Internet addiction. They were instructed to complete the questionnaire within 40 min and could request assistance from a trainee teacher for clarification. Participants were informed of their right to withdraw at any time before completing the questionnaire. The researchers assessed questionnaire completeness, considering mostly incomplete responses as invalid.

### 2.2 Measurements

#### 2.2.1 Psychological maltreatment scale

Emotional abuse was assessed using the Chinese version of Psychological Maltreatment Scale (PMS) developed by Pan et al. (2010). The PMS includes 23 items across the following five domains: terrorizing (e.g., “My parents shouted at me”), derogation (e.g., “My parents listed my shortcomings in front of others”), neglect (e.g., “My parents do not care about the changes in my grades”), interference (e.g., “My parents eavesdrop on my phone calls”), and connivance, encompassing items 22 and 23 (“My parents will not deal with my misbehavior (e.g., fighting, stealing, etc.)” and “My parents do not forbid me to play cards and gamble”). Due to the prevalence of authoritarian parenting style among Chinese parents (Ning, 2022) and the rarity of primary school students in China engaging in activities such as playing cards and gambling, the connivance dimension was not included in this study. A 5-point Likert scale was used, where 0 = “none,” 1 = “rarely,” 2 = “sometimes,” 3 = “often,” and 4 = “always.” Higher scores on the scale indicated a higher level of emotional abuse experienced by the child. In this study, the Cronbach’s alpha coefficients for the PMS and its subscales were as follows: PMS (0.88), terrorizing subscale (0.83), derogation subscale (0.77), neglect subscale (0.61), and interference subscale (0.64). The results of the confirmatory factor analysis (CFA) showed that the PMS model had a good fit, as the chi-square/degree of freedom (χ^2^/df) was 2.78, the Root Mean Square Error of Approximation (RMSEA) was 0.06, the Standardized Root Mean Square Residual (SRMR) was 0.04, the Comparative Fit Index (CFI) was 0.92, and the Three-Function Index (TFI) was 0.90.

#### 2.2.2 Difficulties in emotion regulation scale

Emotional dysregulation was assessed using the Difficulties in Emotion Regulation Scale (DERS), which is a 36-item self-report questionnaire designed by Gratz and Roemer (2004) and has been widely used in assessing adolescents (Mo et al., 2018). The six subscales included impulse control difficulties (Impulse, e.g., “When I’m upset, I lose control over my behaviors”), limited access to emotional regulation strategies (Strategies, e.g., “When I’m upset, I believe that wallowing in it is all I can do”), lack of emotional clarity (Clarity, e.g., “I am confused about how I feel”), difficulties in engaging in goal-directed behavior (Goals, e.g., “When I’m upset, I have difficulty focusing on other things”), nonacceptance of emotional responses (Nonaccept, e.g., “When I’m upset, I become angry with myself for feeling that way”), and lack of emotional awareness (Aware, e.g., “I pay attention to how I feel”). As reverse scoring can potentially impact the reliability and validity of the scale (Rodebaugh et al., 2007; Lee et al., 2016; Venta et al., 2022), the aware dimension requiring reverse scoring was excluded in subsequent analyses. Responses were rated on a 5-point Likert scale ranging from 1 = “never” to 5 = “always,” with higher scores suggesting greater problems with emotional regulation. The Cronbach’s alpha coefficients for the overall DERS and its subscales were as follows: DERS (0.93), impulse subscale (0.87), strategies subscale (0.81), clarity subscale (0.83), goals subscale (0.77), and nonaccept subscale (0.61). The CFA results showed that this scale had an acceptable model fit (χ^2^/df = 3.00, RMSEA = 0.06, SRMR = 0.05, CFI = 0.92, TFI = 0.91).

#### 2.2.3 Regulatory emotional self-efficacy scale

Regulatory emotional self-efficacy (RESE) was assesses using the Chinese version of Regulatory Emotional Self-Efficacy Scale developed by Caprara et al. (2008) and revised by Shufeng et al. (2009). This scale consisted of 12 items across three dimensions: efficacy in regulating positive emotions (e.g., “I feel good about myself when I achieve my goals”), efficacy in regulating depression/pain emotions (e.g., “In the face of sharp criticism, I was able not to lose heart”), and efficacy in regulating angry emotions (e.g., “When I am angry, I can avoid flying into a rage”). The items were assessed on a 5-point Likert scale ranging from “strongly disagree” to “strongly agree.” Possible scores ranged from 12 to 60, with higher scores reflecting greater self-efficacy in emotional regulation. The Cronbach’s alpha coefficients for this scale and its subscales in the present study were as follows: RESE (0.84), positive emotions subscale (0.78), depression/pain emotions subscale (0.76), and anger emotions subscale (0.73). The CFA results revealed that the scale had a good model fit (χ^2^/df = 2.69, RMSEA = 0.06, SRMR = 0.04, CFI = 0.96, TFI = 0.95).

#### 2.2.4 Internet addition test

Internet addiction was assessed using the Internet Addiction Test (IAT) developed by Young (1999). Fernández-Villa et al. (2015) divided this test into two domains: emotional investment and time management and performance. The scale included 20 items rated on a 5-point Likert scale: 1 = “none,” 2 = “rarely,” 3 = “sometimes,” 4 = “often,” and 5 = “always.” Total scores ranged from 20 to 100, with higher scores indicating a greater risk of Internet addiction. The Cronbach’s alpha coefficients for IAT and its subscales in this study were as follows: IAT (0.87), emotional investment subscale (0.85), and time management subscale (0.74). The CFA results showed that the scale had a good model fit (χ2 /df = 2.43, RMSEA = 0.05, SRMR = 0.04, CFI = 0.93, TFI = 0.92).

### 2.3 Statistical analysis

IBM SPSS software version 22.0 and Mplus 8.3 were used for all statistical analyses. Pearson’s correlation analysis was employed to explore the relationships among emotional abuse, emotional dysregulation, regulatory emotional self-efficacy, and Internet addiction. CFA was conducted to assess the validity of the scales using Mplus 8.3. Harman’s one-way test was employed to assess common method bias. The chain-mediating effect was analyzed using Mplus 8.3 with 5,000 bootstrap samples. Statistical significance was considered when the 95% confidence interval did not include 0.

## 3 Results

### 3.1 Common method biases test

The results of Harman’s one-way test identified 18 factors with eigenvalues greater than 1, and the first factor explained only 19.74% of the variance, which was significantly lower than the critical value of 40%. This indicates that there was no significant common method bias.

### 3.2 Preliminary correlation analyses

As shown in Table 2, Pearson’s correlation analysis revealed that emotional abuse was positively correlated with Internet addiction (*r* = 0.37, *p* < 0.001) and emotional dysregulation (*r* = 0.43, *p* < 0.001) and negatively with regulatory emotional self-efficacy (*r* = −0.26, *p* < 0.001). Additionally, emotional dysregulation and regulatory emotional self-efficacy had opposite correlations with Internet addiction, while emotional dysregulation positively correlated (*r* = 0.41, *p* < 0.001) and regulatory emotional self-efficacy negatively correlated (*r* = −0.28, *p* < 0.001).

**Table 2:** Means, standard deviations, and correlations of all variables (*N* = 556).

Variables	1	2	3	4
1. Emotional abuse				
2. Internet addiction in children	0.37***			
3. Emotional dysregulation	0.43***	0.41***		
4. Regulatory emotional self-efficacy	−0.26***	−0.28***	−0.29***	
*M*	15.41	32.31	50.91	43.43
*SD*	11.93	10.25	19.23	8.59

***p < 0.001.

Furthermore, emotional dysregulation was negatively correlated with regulatory emotional self-efficacy (*r* = −0.29, *p* < 0.001).

### 3.3 The chain mediation model

**Figure 2:**
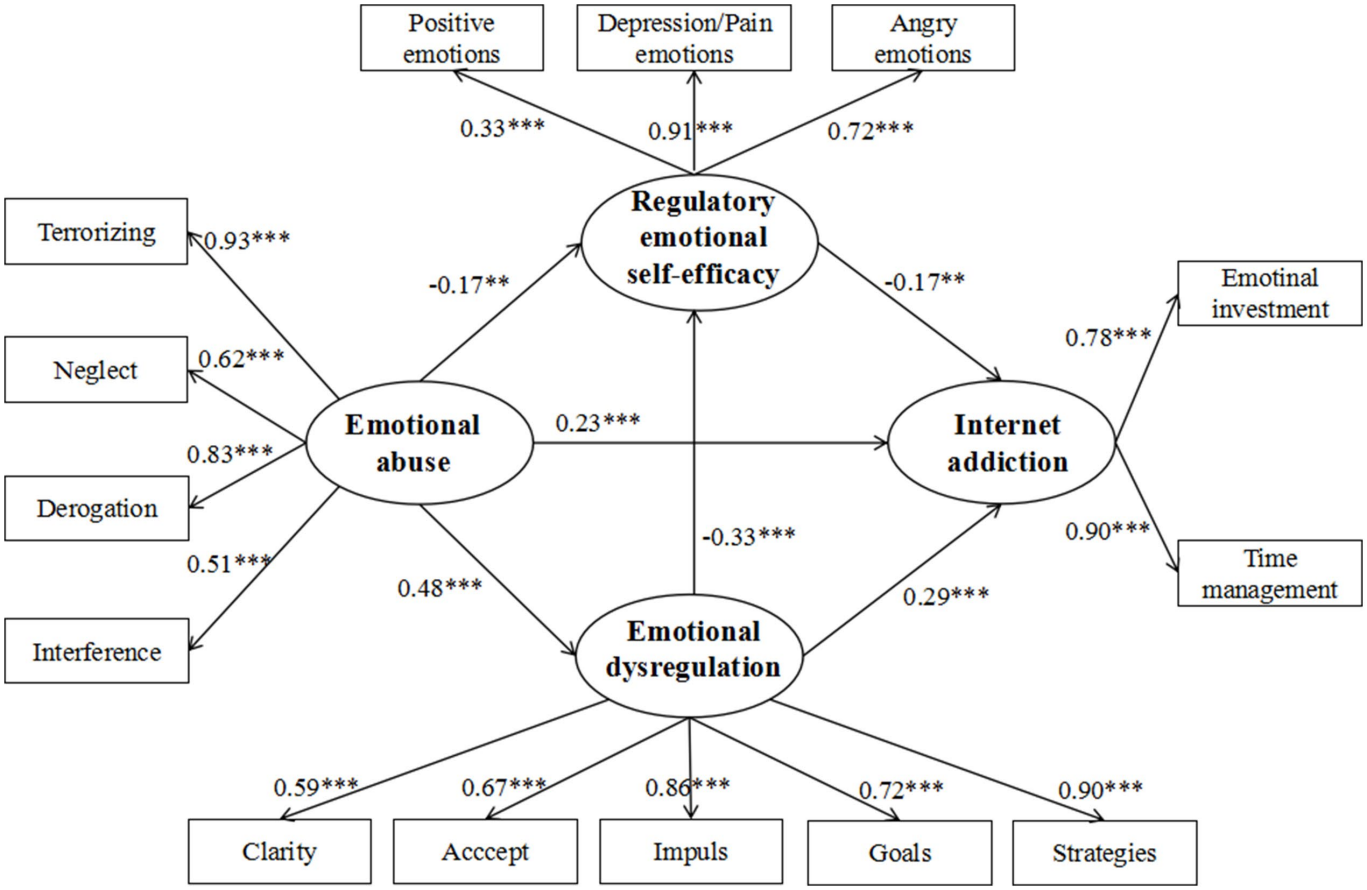
The chain-mediating model linking emotional abuse and Internet addiction through emotional dysregulation and regulatory emotional self-efficacy. **p < 0.01, ***p < 0.001.

The sequential mediation model demonstrated excellent fit indices (χ^2^/df = 2.23, RMSEA = 0.05, SRMR = 0.04, CFI = 0.97, TLI = 0.97). As shown in Figure 2, emotional abuse had a significant positive effect on emotional dysregulation (β = 0.48, *p* < 0.001) and Internet addiction (β = 0.23, *p* < 0.001), and a significant negative effect on regulatory emotional self-efficacy (β = −0.17, *p* < 0.01). Emotional dysregulation positively (β = 0.29, *p* < 0.001) and regulatory emotional self-efficacy negatively predicted Internet addiction (β = −0.17, *p* < 0.01). Additionally, regulatory emotional self-efficacy was significantly affected by emotional dysregulation (β = −0.33, *p* < 0.001).

The mediation effect analysis indicated that the indirect effects paths were significant. Three indirect mediating effect paths were examined: (1) emotional dysregulation partially mediated the relationship between emotional abuse and Internet addiction (indirect effect = 0.14, SE = 0.04, 95% CI = [0.072, 0.210]), (2) regulatory emotional self-efficacy partially mediated the relationship between emotional abuse and Internet addiction (indirect effect = 0.03, SE = 0.02, 95% CI = [0.007, 0.067]), and (3) emotional dysregulation and regulatory emotional self-efficacy mediated the relationship between emotional abuse and Internet addiction in series (indirect effect = 0.02, SE = 0.01, 95% CI = [0.008, 0.053]). As the bootstrap 95% CI did not contain 0, all the three paths reached a significant level, explaining 33.33, 7.14, and 4.77% of the total effect, respectively (see Table 3). Supplementary analysis including demographic variables such as age, gender, grade, only child status, and parental education level revealed no significant changes in any of the path coefficients in the model.

**Table 3:** Analysis of the mediating effect of emotional dysregulation and regulatory emotional self-efficacy.

Paths	Effect	95%CI		Effect ratio
		Low	High	
Direct effect of emotional abuse on Internet addiction in children	0.23	0.107	0.349	54.76%
Emotional abuse → ED → IA	0.14	0.072	0.210	33.33%
Emotional abuse → RESE → IA	0.03	0.007	0.067	7.14%
Emotional abuse → ED → RESE → IA	0.02	0.008	0.053	4.77%
Total indirect effect	0.19	0.128	0.260	45.24%
Total effect of emotional abuse on Internet addiction in children	0.42	0.306	0.522	

ED, emotional dysregulation; RESE, regulatory emotional self-efficacy; IA, Internet addiction.

## 4 Discussion

Given the severe physiological, mental and social consequences of Internet addiction for children (Putra et al., 2023) and the high potential incidence of emotional abuse in Chinese families, it is crucial to explore the impact of emotional abuse on Internet addiction in Chinese children and the underlying mechanism. As expected, the results of this study reveal that emotional abuse positively predicts children’s Internet addiction (Hypothesis 1); emotional dysregulation and regulatory emotional self-efficacy independently mediate the relationship between emotional abuse and Internet addiction (Hypothesis 2); and these two mediators also have a chain mediation effect in the relationship between emotional abuse and children’s Internet addiction because emotional dysregulation negatively affects regulatory emotional self-efficacy (Hypothesis 3).

### 4.1 The direct impact of emotional abuse on Internet addiction in children

This study reveals that emotionally abused children are prone to developing Internet addiction. This is an important addition to previous findings that adults and adolescents who have experienced high levels of emotional abuse in childhood are more likely to have Internet addiction (Xu and Zheng, 2022; Arslan, 2017). However, emotional abuse in childhood is assessed in a retrospective way in these studies, which may introduce significant recall bias that could result in underreporting or overreporting of emotional abuse (Danese, 2020; Yu et al., 2024). Unlike adolescents or adults, children may not have this problem when reporting emotional abuse experiences over a short period of time (Danese, 2020). More importantly, previous studies have shown that emotional abuse in childhood has a long-term impact on Internet addiction in their adolescence period or even in adulthood, while our results have demonstrated that Internet addiction can also act as an immediate response to emotional abuse in children. This finding aligns with the I-PACE theory, which emphasizes that negative childhood experiences, such as emotional abuse, can lead to the accumulation of negative emotions and subsequently the development of Internet addiction (Brand et al., 2019). This underscores the importance of addressing emotional abuse early in life to mitigate its potential impact on the development of Internet addiction.

The results suggest that emotional abuse and its negative consequences significantly impact children within the context of Chinese culture. Chinese children may be particularly susceptible to emotional abuse for several reasons. First, some forms of emotional abuse may be misinterpreted and used by parents under the guise of promoting child development, especially given the prevalence of authoritarian parenting styles (Ning, 2022). Second, children in China are often taught to uphold traditional values, such as filial piety and respect for authority, which encourages them to suppress their true feelings and conform to their parents’ expectations (Bedford and Yeh, 2019; Fu et al., 2007). Additionally, emotional abuse is often stigmatized in public discourse, which leads to inadequate psychological support and legal protection for affected children. In this context, emotionally abused children may turn to the Internet as a means of coping with unmet emotional needs and relieving negative emotional states resulting from parental frustration. Therefore, our study offers a cultural perspective on the link between emotional abuse and Internet addiction. Furthermore, the results suggest that, implementing measures at the levels of parents, children, and society to reduce the risk of emotional abuse is crucial for preventing children’s Internet addiction and mitigating the negative consequences of emotional abuse in China.

### 4.2 The mediating roles of emotional dysregulation and regulatory emotional self-efficacy

Emotional dysregulation plays a mediating role between emotional abuse and Internet addiction in children. This aligns with prior research indicating that emotional neglect significantly impairs emotional intelligence, including emotional understanding, management, and coping strategies, thereby predisposing adolescents to smartphone addiction (Sun et al., 2019). This finding offers a new understanding of how emotional abuse contributes to Internet addiction through the compromised emotional regulation abilities in children. Children who undergo emotional abuse may develop negative feelings, including loneliness, anxiety, and depression. These adverse emotions can subsequently lead to difficulties in regulating their emotions (Cui and Liu, 2020). This also aligns with the I-PACE model, which posits that emotional abuse may alter children’s coping styles for negative emotions, potentially leading to Internet addiction, and the Internet Compensation Theory, which posits that children experiencing high levels of emotional abuse may turn to excessive Internet use as a coping mechanism in their struggle to manage the negative emotional states as a result of parental emotional abuse (Valkenburg and Peter, 2009).

This study also highlights regulatory emotional self-efficacy as a crucial mediator in the relationship between emotional abuse and Internet addiction in children. Compared to emotional dysregulation that is a state-like construct and is expected to vary from situation to situation, regulatory emotional self-efficacy is more of a trait-like construct that is fairly consistent over time. Thus, this finding provides evidence that emotional abuse has not only state-like but also trait-like impact on children’s emotional coping. Specifically, emotional abuse leads children to have lower self-confidence in their ability to regulate emotions, potentially predisposing them to Internet addiction. The impact of emotional abuse on regulatory emotional self-efficacy resembles the psychological phenomenon of gaslighting, whereby the perceptions of victims are manipulated and their confidence in their thoughts and capabilities are undermined (Akdeniz and Cihan, 2024). When their confidence in emotional regulation diminishes, emotionally abused children may seek refuge in Internet addiction rather than confronting negative emotions caused by emotional abuse. This argument is supported by the Self-efficacy Theory, which states that one’s confidence in emotional abilities influences his or her efforts to regulate negative emotions, potentially lowering the risk of Internet addiction (Bandura and Adams, 1977). Therefore, interventions aimed at enhancing regulatory emotional self-efficacy, correcting distorted self-beliefs about emotional abilities and fostering self-confidence in emotional regulation, have the potential to effectively mitigate Internet addiction risk in emotionally abused children.

### 4.3 The sequential mediation roles of emotional dysregulation and regulatory emotional self-efficacy

This study shows that emotional dysregulation and regulatory emotional self-efficacy play serial mediating role in the relationship between emotional abuse and Internet addiction in children. Specifically, children repeatedly exposed to emotional abuse will find it difficult to regulate their negative emotions arising from parental emotional abuse, leading to a decline in their confidence in effective management of emotions and consequently increasing the risk of Internet addiction. This finding aligns with the I-PACE model, which posits that emotional abuse leads to changes in affective states, followed by cognitive shifts, ultimately resulting in addictive behavior.

This sequential mediation can also be explained by the theory of learned helplessness, where abused children may repeatedly fail to cope with negative emotions due to their limited emotional regulation abilities, resulting in decreased confidence in managing these emotions (Maier and Seligman, 1976). As suggested by Social Cognitive Theory, it takes experience and time to develop self-efficacy beliefs about one’s abilities (Bandura, 1999). This sequential mediation provides additional support for this theoretical framework, demonstrating that emotional abuse not only impairs emotional regulation in children from a state-based perspective, but also shapes their trait-like cognitive beliefs about their emotional capabilities due to repeated failures. This result suggests that, even when the development of emotional regulation is impeded in abused children (Jacobs and Nader-Grosbois, 2020), enhancing their regulatory emotional self-efficacy—by altering their cognitive beliefs about their emotional abilities—can mitigate the impact of emotional abuse on Internet addiction.

### 4.4 Practical implications

The current study has important practical implications for preventing Internet addiction in Chinese children. First, we emphasize the importance to eliminate emotional abuse during parenting to diminish the risk of Internet addiction. Accordingly, developing family education programs aimed at helping parents reduce authoritarian parenting and emotional abuse may effectively decrease emotional abuse (Brodski and Hutz, 2012), thereby preventing children from developing Internet addiction. These family education programs may include teaching parents to identify and adjust behaviors related to emotional abuse, replace harmful practices with positive and constructive methods to encourage their children’s growth, and regulate negative emotions and parenting stress. To more effectively assess the impact of parent education, we can compare changes in parental perceptions and knowledge of emotional abuse, abusive behaviors, and children’s perceptions of emotional abuse before and after the program. In addition, the government or social welfare organizations should establish behavioral norms and public education campaigns to raise awareness about emotional abuse and reduce discrimination against it and its negative consequences. The state can also implement relevant laws to regulate emotional abuse, especially when parents are unaware of their own emotionally abusive behaviors.

Second, since abused children often struggle to recognize emotional abuse and express and cope with their feelings within Chinese culture, as evidenced by the mediating role of emotional dysregulation, it is crucial for psychological educators to enhance emotional regulation skills among these children. This involves improving their ability to recognize and respond to emotional abuse, manage the negative emotions it causes, and communicate effectively with their parents. After emotion regulation skills training, we can assess children’s emotional regulation abilities through situational simulations of emotional abuse scenarios. Additionally, given that cognitive development is limited during childhood and abused children often have impaired emotional regulation, improving their emotional skills may not yield immediate effects (Kumari, 2020). As suggested by the chain-mediating role of regulatory emotional self-efficacy, it is crucial for abused children, whose emotional regulation is impaired or still developing, to enhance their regulatory emotional self-efficacy by cultivating confidence in their emotional regulation abilities. Parents and teachers can teach children to use positive psychological cues to encourage and affirm their emotional regulation abilities, or create simple emotional regulation scenarios to help children gain successful emotional regulation experiences.

### 4.5 Limitations and implications

This study has several limitations. Firstly, more longitudinal studies with different time lags are needed to demonstrate the long-term impact of emotional abuse on Internet addiction in children. Secondly, although previous studies have suggested that Internet addiction is a common problem faced by most students in China (Chen et al., 2021; Wu et al., 2022), the participants in this study were only recruited from three schools in a specific province in central China. Therefore, the research sample was limited to participants from a particular region. Expanding the sample size to include participants from a broader range of regions, incorporating areas with diverse cultural and economic characteristics, and explicitly examining the influence of regional factors will help improve the generalizability of the results in future research. Thirdly, we have focused on children’s perception of emotional abuse, which appear to be a more proximal predictor of Internet addiction compared to the actual emotional abuse perpetrated by their parents. Given the possible discrepancy between perceived and actual emotional abuse within Chinese culture, it is necessary to assess the actual perpetration of emotional abuse by their parents. Additionally, the results obtained from the questionnaires are dependent on the subjective responses of the participants, which are susceptible to biases such as memory bias and social desirability bias. For example, children might intentionally conceal or distort their true experiences of emotional abuse or Internet addiction in order to meet the expectations of parents or teachers, thus affecting the accuracy and dependability of the data. To mitigate this limitation, future studies should employ a variety of methods to measure emotional abuse, incorporating additional indicators such as the observation and recording of parent–child interactions or the inclusion of third-party assessments. Moreover, utilizing physiological measurement tools, such as heart rate variability monitors or skin conductance response sensors, could offer deeper insights into children’s physiological responses when exposed to emotionally charged stimuli, such as video clips designed to evoke emotions like sadness or anger. Also, future studies may benefit from incorporating brain function information of emotional dysregulation and regulatory emotional self-efficacy of emotionally abused children. Lastly, the differential impacts of emotional abuse on different sub-types of Internet addiction in children remain to be elucidated in future studies. For instance, emotional abuse may influence online social media addiction via emotional pathways (Worsley et al., 2018) and Internet gambling addiction via cognitive pathways (Dong et al., 2017).

## 5 Conclusion

This study contributes to the Internet addiction literature by investigating the impact of emotional abuse on Internet addiction through the sequential mediation of emotional dysregulation and regulatory emotional self-efficacy in a sample of Chinese children. The findings illustrate the immediate influence of emotional abuse on children’s Internet addiction and provide a novel explanation of the relationship between these factors within the context of Chinese culture. A crucial strategy to decrease children’s Internet addiction is to reduce emotional abuse in parenting. Emotional dysregulation and emotional self-regulation efficacy serve as chain mediators in the relationship between emotional abuse and Internet addiction in children. Specifically, emotional abuse not only disrupts children’s state-like ability to regulate their emotions but also affects their trait-like perception of emotional regulation, thereby leading to Internet addiction. The sequential mediation effects suggest that enhancing the emotional regulation confidence of children who have been emotionally abused, despite their current limitations in this area, may still offer protection against developing Internet addiction.

## Data Availability

The raw data supporting the conclusions of this article will be made available by the authors, without undue reservation.
